# One Cluster per Condition Is Not a Valid Design in Cluster-Randomized Trials. Comment on Wang et al. The Effect of Different Physical Exercise Programs on Physical Fitness among Preschool Children: A Cluster-Randomized Controlled Trial. *Int. J. Environ. Res. Public Health* 2023, *20*, 4254

**DOI:** 10.3390/ijerph20176673

**Published:** 2023-08-29

**Authors:** Yasaman Jamshidi-Naeini, Armando Pena, Abu Bakkar Siddique, Andrew W. Brown, David B. Allison

**Affiliations:** 1 Department of Epidemiology and Biostatistics, Indiana University School of Public Health-Bloomington, Bloomington, IN 47405, USA; 2 Department of Health and Wellness Design, Indiana University School of Public Health-Bloomington, Bloomington, IN 47405, USA; 3 Department of Biostatistics, University of Arkansas for Medical Sciences, Little Rock, AR 72205, USA; 4 Arkansas Children’s Research Institute, Little Rock, AR 72202, USA

Wang et al. [1] conducted a study to “determine the effectiveness of and the differences between different physical exercise programs in improving preschool children’s physical fitness”. The study is identified as a cluster-randomized controlled trial (cRCT) in the article. As described in the article, preschool children were recruited from five kindergartens, and groups were randomized by kindergarten to four interventions and one control group. That is, there was one kindergarten assigned to each of the five study groups, rendering the study design invalid for accurately estimating the causal effect of treatment assignment.

In cRCTs, the presence of correlated data within each cluster is expected and often observed, which means that assumptions of independence for errors (or model residuals) across study participants within clusters may not hold. Moreover, in studies where clusters, rather than individuals, are assigned to study conditions, individuals are nested within clusters, which creates a hierarchical data structure [2]. Hence, the available degrees of freedom for testing the intervention effect rely on the number of individual participants and the number of clusters allocated to each condition [3]. In a purported cRCT with one cluster allocated to each of the conditions, such as Wang et al.’s study, it is not possible to estimate the variation attributed to cluster membership separately from the variation attributed to treatment assignment. Therefore, intervention effects and uncertainty cannot be estimated using such a design [4] and the trial should not be labeled as a cRCT.

Although assigning clusters (i.e., kindergartens) instead of individuals can be justified in Wang et al.’s study, the confounding of treatment and clusters by having one cluster per condition does not allow the level of causal inference about the intervention effect that can be drawn from cRCTs. Therefore, the study should be labeled as a quasi-experimental study with numerical results interpreted as evidence of associations to clarify the weaker causal inferences from quasi-experimental compared to randomized designs [5].
